# The role of acetic acid in orthopaedic surgery

**DOI:** 10.1177/17504589211015629

**Published:** 2021-07-26

**Authors:** Yousuf Hashmi, Andrew Kailin Zhou, Anam Jawaid, Anli Yue Zhou, Vianca Shah, Azeem Thahir, Matija Krkovic

**Affiliations:** 1 College of Medical and Dental Sciences, University of Birmingham, School of Medicine, Birmingham, UK; 2 Department of Trauma and Orthopaedics, Addenbrookes Major Trauma Unit, Cambridge University Hospitals, Cambridge, UK; 3 School of Clinical Medicine, University of Cambridge, Cambridge, UK; 4 School of Health Sciences, University of Manchester, Manchester, UK; 5 Natural Science Department, University of Cambridge, Cambridge, UK

**Keywords:** Iontophoresis, Debridement, Soft tissue injuries

## Abstract

Acetic acid has become more commonly used in orthopaedic surgery. The purposed roles include biofilm eradication and surgical debridement, postoperative scar reduction and managing soft tissue injuries. Current research is scarce and does not provide conclusive evidence behind acetic acid’s efficacy in orthopaedic procedures such as biofilm eradication or acetic acid iontophoresis in soft tissue injuries. Current literature on acetic acid’s effects on biofilm eradication is composed of in-vitro studies, which do not demonstrate the potential clinical efficacy of acetic acid. Acetic acid iontophoresis is a novel technique which is now more commonly accepted for soft tissues injuries. Our literature search identified calcifying tendonitis of the shoulder, rotator cuff tendinopathy, heel pain syndrome, plantar fasciitis, achilles tendonitis, calcifying tendonitis of the ankle, myositis ossificans and cervical spondylosis as documented clinical uses. In this narrative review, we present the current uses of acetic acid and acetic acid iontophoresis, while evaluating the evidence revolving around its efficacy, benefits and risks.

**Provenance and Peer review:** Unsolicited contribution; Peer reviewed; Accepted for publication 17 April 2021.

## Introduction

Acetic acid (AA) is commonly used in orthopaedic surgery for its aseptic properties (Kavolus et al 2020). Recently, it has been adapted for other uses, depending on the operating surgeon’s preference, which include managing soft tissue injury, reducing postoperative scarring, biofilm eradication (periprosthetic) and debridement (Dardas et al 2014, Tsang et al 2018, Williams et al 2017).

Currently, arthroplasties require thorough debridement as compulsory for treatment of periprosthetic joint infection (PJI), and there have been reports of AA being effective in treating these cases (Williams et al 2017). Furthermore, biofilms, already known to be involved in a variety of chronic infections relevant to orthopaedics, have been recognised as one of the most critical steps to the pathogenesis of periprosthetic joint infections, and evidence has shown AA to be effective in biofilm eradication (Bjarnsholt et al 2015, Tsang et al 2018). Other research has shown AA to be effective in postoperative scar reduction, with acetic acid ionophoresis (AAI) driving the large effort to minimise postoperative scar formation due to the unpredictability and potential for serious scarring (Dardas et al 2014, Qureshi & Orgill 2012).

The current literature has very little evidence on the clinical effectiveness of AA and its roles in orthopaedic surgery (Dardas et al 2014, Tsang et al 2018, Williams et al 2017). There has also been much controversy around the role of AA in orthopaedics, as there is unclear guidance for surgeons and currently its use is considered ‘off-licence’ (Medicines & Agency HpR 2009).

In this narrative review, we discuss the current licensed and off-licenced uses of acetic acid while evaluating its associated risks. We have summarised the key indications and provided a guideline of the use of AA in orthopaedic surgery.

## Methods

A thorough literature search was performed in PubMed, Medline, Embase and Cochrane databases using the keywords (acetic acid) and (orthopaedic OR orthopedic). These search terms were used because there is very little literature on this; therefore, we tried to be broad and ensure that relevant papers were included.

All abstracts of retrieved articles were reviewed by four of the authors to ensure they were applicable. Inclusion criteria included articles which discussed the use of AA in orthopaedic surgery and all other articles which discussed the use of AA outside of orthopaedic surgery were excluded.

## Debridement and biofilm eradication (A tool against periprosthetic joint infection)

Wound healing is critical postsurgery, and complications such as periprosthetic joint infections are increasingly resistant to standard antibiotic therapy (Trampuz et al 2004). This growing redundancy of traditional methods to combat the poor prospect of periprosthetic joint infections has led to the popularisation of agents, such as AA, in debridement and biofilm eradication, replacing typically used antimicrobial substances (Tsang et al 2018). Currently, the literature is scarce; however, there is encouraging evidence with regard to the use of AA in biofilm eradication which has the potential for clinical use where standard management has not been effective. There are a couple of ambiguities in the literature with regard to the specifics of clinical AA use such as optimum AA concentration or duration of soak. This calls for further clinical research on AA and we have provided a few recommendations below on exploring the clinical research on AA in debridement.

The use of AA as a chemical debridement agent is poorly documented in literature. AA was used in wound treatment and has been known since the times of Hippocrates (Johnston & Gaas 2006). More recently, AA soaks have been described as an adjuvant chemical debridement tool to treat PJIs (Williams et al 2017). Bjarnsholt et al (2015) investigated the in-vitro use of AA in isolation and in combination with negative pressure wound therapy (NPWT). Complete eradication of *Staphylococcus aureus* was obtained when using 1.0% AA, and likewise with either 0.5% or 1.0% AA for *Pseudomonas aeruginosa* biofilms (Bjarnsholt et al 2015). Whereas hydrochloric acid (HCL), as a control, had no effect on *P. aeruginosa* biofilms and only achieved partial eradication on *S. aureus* biofilms (Bjarnsholt et al 2015). Maximal antimicrobial properties of AA was observed at a pH of 4.76 and lower; meanwhile, the use of HCL in the same conditions did not result in antimicrobial activity (Bjarnsholt et al 2015). Furthermore, Bjarnsholt et al (2015) demonstrated that a significant antimicrobial effect is seen at the equilibrium pH of acetic acid, which is unlike other organic acids such as HCL. This suggests that the non-dissociated form is responsible for killing organisms, and not the proton dissociation of AA (Agrawal et al 2017, Bjarnsholt et al 2015). To verify that these same biofilm model systems were tolerant to antibiotics, but susceptible to AA, suspensions of tobramycin, ciprofloxacin and colistin were added individually into each biofilm model investigated and none of these were shown to have a significant effect on bacteria eradication (Bjarnsholt et al 2015).

Looking synergistically, there is potential for a combinatory treatment of AA with antibiotics. The same study by Bjarnsholt et al (2015) showed that the combination of AA and the antibiotic bramycin resulted in an enhanced antibacterial effect.

A similar study evaluated the potential for AA using clinically relevant treatment times (ten or 20min) in the eradication of methicillin-susceptible *Staphylococcus aureus* (MSSA) (Tsang et al 2018). The minimum biofilm eradication concentration of AA was greater than the safety threshold of 5%, but if a clinically acceptable, lower concentration of 5% was used, 96.1% of biofilm-associated MSSA was eradicated following 20min of treatment (Tsang et al 2018). This provides potential for the use of AA as a non-toxic, topical debridement adjunct (Bjarnsholt et al 2015, Tsang et al 2018).

Current strategies in preventing bacterial biofilm infections include perioperative antibiotic prophylaxis, or suppressive antibiotic treatment for established, treatment-resistant biofilms (Bjarnsholt et al 2015). Bacterial biofilms, however, in such cases of periprosthetic joint infection, are known to be resistant towards antibiotics and other antimicrobial agents (Ravi et al 2016). Evidence for AA as a possible therapy for near complete eradication of both gram-positive and gram-negative biofilms, is extremely promising; therefore, AA has the potential for clinical use where standard has not been effective (Bjarnsholt et al 2015).

While the scarce literature did show encouraging results with regard to the use of AA in the eradication of biofilms, there is minimal evidence with regard to identifying an optimum concentration or duration of soak, thereby leaving many ambiguities. This calls for further randomised controlled studies needed to evaluate its clinical value. More investigation is required into the effects of AA on a greater variety of bacterial species, as well as a thorough clinical evaluation on the use of 5% AA at different soak durations, looking directly at the safety profile, surgical outcomes when used in the treatment of periprosthetic joint infections and patient tolerance.

## Acetic acid iontophoresis for soft tissue injury

Acetic acid iontophoresis (AAI) is a process allowing the penetration of ionized molecules across or into a tissue, by applying a low electric current. AAI applies acetic acid, an inorganic anion, to the cathode and upon application of the current, the ionised molecules migrate to the anode. This passage of molecules occurs through the skin, primarily through hair follicles and sweat gland canals (Kachewar & Kulkarni 2013).

When utilised in this manner, AA is effective in the management of ossifying conditions due to its acidic properties (Gard & Ebaugh 2010, Kachewar & Kulkarni 2013). The calcification mainly consists of hydroxyapatite crystals which regress upon application of the acid (Kachewar & Kulkarni 2013). This is due to the crystals being insoluble in water but soluble in acidic pH environments (Kachewar & Kulkarni 2013).

The utilisation of AAI has been reported in multiple pathologies, as it is a safe, simple and inexpensive technique (Fernández Cuadros et al 2016). Kilfoil et al (2014) have reported bilateral Achilles tendinitis being treated with AAI therapy of 4% AA (Phoresor PM900 at a setting of 2.0mA DC current) for 20min for five times over two weeks. The positive electrode was placed on the skin of the midshaft of the lateral fibula and the negative electrode was placed the insertion of the Achilles tendon on the heel bilaterally (Kilfoil et al 2014). However, since there is no official guidance on the use of AAI therapy, other studies have reported different concentrations of AA and different durations (Costa & Dyson 2007, Gard & Ebaugh 2010). This calls for further research investigating the optimal therapeutic concentrations, duration and frequency of iontophoresis.

Our literature search identified calcifying tendonitis of the shoulder, rotator cuff tendinopathy, heel pain syndrome, plantar fasciitis, Achilles tendonitis, calcifying tendonitis of the ankle, myositis ossificans and cervical spondylosis as documented uses (Bagnulo & Gringmuth 2014, Fernández Cuadros et al 2016, Japour et al 1999, Osborne & Allison 2006, Perron & Malouin 1997). When treating calcifying tendonitis and Achilles tendonitis, AAI therapy is coupled with therapeutic ultrasound in some cases, possibly indicating improved outcomes when used as a combined treatment (Fernández Cuadros et al 2016, Perron & Malouin 1997).

Calcifying tendonitis of the shoulder is one of the most extensively documented uses of AAI (Leduc et al 2003, Perron & Malouin 1997). Perron and Malouin (1997) and Leduc et al (2003) both carried out randomised control trials, where the effectiveness of AAI was investigated in the pathology. Perron and Malouin (1997) looked at the use of AAI followed by ultrasound, while Leduc et al (2003) looked at the therapeutic effects of AAI only on calcifying tendonitis. In both studies, AAI led to improvement of the condition; however, only results from the latter study were significant (P < 0.001) (Leduc et al 2003, Perron & Malouin 1997). The trial by Perron and Malouin (1997), with 21 participants, has a high risk of bias, as well as both detection and performance bias, and imprecision in data collection, as evaluated by Page et al (2016). Looking at the similar study by Leduc et al (2003), there was 25% attrition, possibly biasing results in favour of one of the study groups, reducing the quality of evidence due to high attrition rate and again imprecision in collection of data when evaluated by Page et al (2016). Most pertinently, both studies were carried out more than two decades ago, and so questions regarding relevance are raised. Thus, the research on this topic requires updating before contemporary conclusions are able to be drawn on the use of AAI in calcifying tendonitis of the shoulder.

Another randomised controlled trial looked at the use of AAI, compared to dexamethasone iontophoresis and placebo, when combined with LowDye taping in plantar fasciitis patients (plantar arch support) (Osborne & Allison 2006). Six treatment doses of iontophoresis were administered to a sample of 31 double-blinded participants over a two-week period (Osborne & Allison 2006). The best clinical results, after four weeks, were found in the AA group (Osborne & Allison 2006). Interestingly, AAI relieved morning stiffness in patients, while dexamethasone iontophoresis relieved overall pain, pointing to the possibility of different combinations of interventions necessary in order to tackle differential primary complaints (Osborne & Allison 2006). Further research is required in this field.

A cohort study by Japour et al (1999) investigated the use of AAI in managing heel pain syndrome in a sample of 35 patients. While the study reported significant results, with AAI treatment causing patient rating of pain to drop significantly, from 7.5 out of 10 to 1.8 out of 10 over a four-year period, the key flaw in this design is the absence of a control group (Japour et al 1999). Without a baseline comparator, it is impossible to say with confidence that these results are due to the effects of AAI alone. Furthermore, there is an absence on details of AAI technique, such as concentration and duration of iontophoresis, and no elaborations on whether blinding took place within the study.

In a prospective cohort study of ten patients with Achilles tendonitis, patients were treated with firstly 10min of AAI, and then 5min of ultrasound over the calcification, for an average of 21 sessions (Fernández Cuadros et al 2016). Treatment showed a statistically significant improvement of pain, as was measured by a visual analogue scale, with pain scores dropping from 7.9 to 2.8 and calcification size also reducing (Fernández Cuadros et al 2016). Limitations of this trial most notably include the lack of a control group, which is explained by the authors as being due to a limited number of cases. Additionally, since this was a non-randomised control trial, randomisation was not possible (Fernández Cuadros et al 2016).

The use of AAI in other conditions, namely Myositis ossificans, cervical spondylitis, is all currently only supported by weak evidence in literature; case reports have all documented improvements in pain and reductions in the size of calcifications; however, more research is required in order to fully understand the exact mechanism of AAI as analgesic therapy (Bagnulo & Gringmuth 2014, Wieder 1992). Avenues of future research include investigating optimal therapeutic concentrations, duration of iontophoresis, statistical significance of the benefits of AAI when combined with ultrasound therapy and the differential effect of AAI depending on patient factors.

## AAI in scar reduction

Postoperative scarring is cellular change at the wound site following tissue injury (Qureshi & Orgill 2012). The process begins with surgical incision and seeks to restore skin integrity via activation of several inflammatory pathways. Scars can range from small, unnoticeable lines, through to painful or disfiguring hypertrophic scars. There is a large effort to minimise postoperative scar formation due to the unpredictability and potential for serious scarring (Qureshi & Orgill 2012). The current management of postoperative scarring includes adequate wound hydration via foam dressings, topical cream application, silicone sheeting, pressure therapy, corticosteroid injection, ultrasound and orthotic intervention (Dorf et al 2010, Son & Harijan 2014).

A retrospective cohort comparison by Dardas et al (2014) analysed a group of 17 patients (23 digits) with recalcitrant scarring following an open trigger finger release. Patients only received AAI treatment if there was a premature plateau in their total active range of motion after receiving occupational therapy; the normal standard of care (Son & Harijan 2014). The study found a statistically significant increase (P < 0.01) in the total active range of motion of patients who underwent the AAI treatment. However, the study had several limitations such as the small sample size and lack of an adequate control cohort. Consequently, a randomised prospective study and further research into the treatment of other scarring sites are still required to provide substantial evidence base.

There is currently no consensus in the literature for the mechanism of action for AA in the context of postoperative scarring. One study identified that exposure to 0.05M AA causes the tightly banded structure of type I collagen fibres to be lost (Yannas et al 1981). As type I collagen is secreted by dermal fibroblasts during scar formation, it is possible that the AAI causes a similar structural change to the fibres at the scar site (Hardy 1989). A randomised controlled trial also highlighted further molecular effects of AA on collagen’s structure (Ohnishi et al 1998). The study found that AA was able to induce swelling of type I collagen fibres and promote a dissociation of the collagen–collagen bonds in a hepatocellular carcinoma nodule. It is therefore likely that AA induces a similar physiological change during scar formation, thus helping to reduce postoperative scarring. More research needs to be done in this area to determine the exact role of AA as a treatment.

## Guidance on using AA in orthopaedic practice (UK)

Currently, the National Institute of Health and Care Excellence (NICE) recommended guidelines for chronic wounds include antimicrobial dressing and advanced wound healing dressings. The guidance from the UK Medicine and Healthcare products Regulatory Agency (MHRA) (2009) determines that AA can be prescribed if all the following are true (MHTA 2009):

1. No alternative medicine that is licensed will not meet the patients’ needs more than AA;
2. The use of AA will be more beneficial for the patient’s needs than any alternative licensed medicine;
3. The surgeon has a sufficient evidence base and experience of using AA to understand its safety and efficacy;
4. The surgeon must take responsibility for prescribing AA and overseeing care of the patient;
5. The surgeon must record that this use of AA is an off-licence use, not common practice and must document the reasons for prescribing and should document a discussion of AA with the patient.

The MHRA also discusses the best practice for communication to discuss the use of AA with the patient (MHRA 2009):

1. The surgeon must provide enough information about AA to enable them to make an informed decision.
2. It may not be necessary to draw attention to the licence when seeking consent for AA for the patient; however, MRHA suggest the doctor to give as much information as they see relevant for the patient or carers.

## Conclusion

AA use potentially offers a plethora of benefits which help resolve significant concerns in orthopaedics. Although AAI has demonstrated benefits in a variety of soft tissue injuries, the evidence within each type of injury is limited, and majority of the evidence is based upon case reports or studies with small sample sizes; therefore, more research is required in order to fully understand the exact mechanism of AAI in the management of soft tissue injuries. Currently, the MHRA has not specified specific guideline with regard to the use of AA in orthopaedic surgery; therefore, surgeons should treat the use of AA as ‘off licence’ and its use should be used in certain parameters. However, the use of AA in orthopaedic surgery has a high upside as it is a cheap, widely available anti-microbial that offers a variety of potential benefits and uses in cases which are resistant to standard treatment. Avenues for future research include investigating optimal therapeutic concentrations, duration of iontophoresis, statistical significance of the benefits of AAI when combined with ultrasound therapy, the differential effect of AAI depending on patient factors, efficacy of AA on a greater variety of bacterial species, clinical evaluation on the use of 5% AA at different soak durations, surgical outcomes when used in the treatment of periprosthetic joint infections, infected metalwork and patient tolerance.

*No competing interests declared*

## Supplemental Material

sj-pdf-1-ppj-10.1177_17504589211015629 - Supplemental material for The role of acetic acid in orthopaedic surgery Click here for additional data file. Supplemental material, sj-pdf-1-ppj-10.1177_17504589211015629 for The role of acetic acid in orthopaedic surgery by Yousuf Hashmi, Andrew Kailin Zhou Anam Jawaid, Anli Yue Zhou, Vianca Shah, Azeem Thahir and Matija Krkovic in Journal of Perioperative Practice
