# Supplementary material for: The role of acetic acid in orthopaedic surgery
Source: J Perioper Pract. 2021 Jul 26;32(6):162–6. doi: 10.1177/17504589211015629 (PMC9149521; doi:10.1177/17504589211015629)

Visual Summary of Acetic Acid in Orthopaedic Surgery

Reported Uses of AA in Orthopaedics

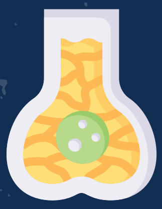

Debridement and Biofilm Eradication

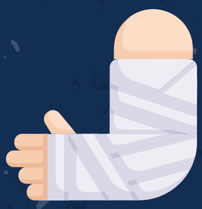

Soft Tissue Injuries

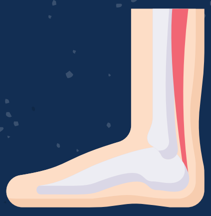

Achilles Tendinitis

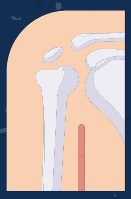

Calcifying Tendinitis

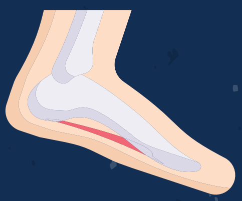

Plantar Fasciitis

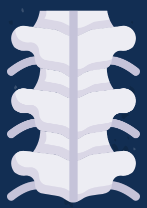

Cervical Spondylitis

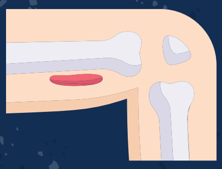

Myositis Ossificans

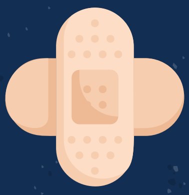

Post-operative Scarring

Recommendations for Clinical AA Use

Biofilm and debridement eradication:
Potential use when standard debridement treatment fails.

Soft tissue injuries:
Consider use when standard management fails

Post-operative scarring:
Consider use when patient improvement plateaus and scarring still symptomatic

Areas for Further Clinical Research

- Different soak durations
- Optimal concentration
- Patient tolerance
- Periprosthetic joint infections
- Infected metalwork

- Duration of AAI
- AAI combined with therapeutic ultrasound
- Different patient factors affecting outcomes

MRHA Guidance on Clinical AA Use

1. Alternative medicine that is licensed will not meet the patients' needs more than AA
2. The use of AA will be more beneficial for the patient's needs than alternative licensed medicine
3. The surgeon has a sufficient evidence base and experience of using AA to understand its safety and efficacy
4. The surgeon must take responsibility for prescribing AA and overseeing care of the patient
5. The surgeon must record that this use of AA is an off-licence use, not common practice and must document the reasons for prescribing, and should document a discussion of AA with the patient

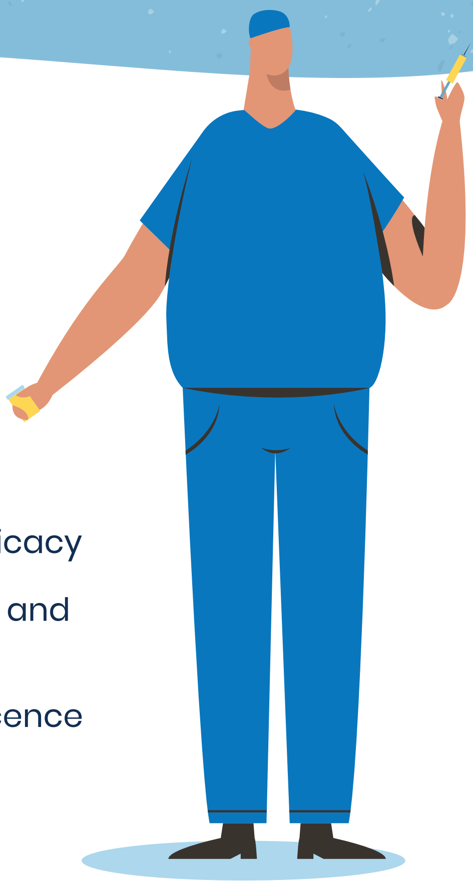

Supplement: sj-pdf-1-ppj-10.1177_17504589211015629 - Supplemental material for The role of acetic acid in orthopaedic surgery [file sj-pdf-1-ppj-10.1177_17504589211015629.pdf]
